# The multiscale 3D convolutional network for emotion recognition based on electroencephalogram

**DOI:** 10.3389/fnins.2022.872311

**Published:** 2022-08-15

**Authors:** Yun Su, Zhixuan Zhang, Xuan Li, Bingtao Zhang, Huifang Ma

**Affiliations:** ^1^School of Computer Science and Engineering, Northwest Normal University, Lanzhou, China; ^2^School of Electronic and Information Engineering, Lanzhou Jiaotong University, Lanzhou, China

**Keywords:** BCI, emotion recognition, EEG, 3D CNN, spatiotemporal features, deep learning

## Abstract

Emotion recognition based on EEG (electroencephalogram) has become a research hotspot in the field of brain-computer interfaces (BCI). Compared with traditional machine learning, the convolutional neural network model has substantial advantages in automatic feature extraction in EEG-based emotion recognition. Motivated by the studies that multiple smaller scale kernels could increase non-linear expression than a larger scale, we propose a 3D convolutional neural network model with multiscale convolutional kernels to recognize emotional states based on EEG signals. We select more suitable time window data to carry out the emotion recognition of four classes (low valence vs. low arousal, low valence vs. high arousal, high valence vs. low arousal, and high valence vs. high arousal). The results using EEG signals in the DEAP and SEED-IV datasets show accuracies for our proposed emotion recognition network model (ERN) of 95.67 and 89.55%, respectively. The experimental results demonstrate that the proposed approach is potentially useful for enhancing emotional experience in BCI.

## Introduction

Automatic EEG-based emotion recognition has been a research hotspot in the field of BCI and human–computer interaction over the past decade. Efficient emotion recognition methods based on EEG can prompt BCI to build a harmonious human-computer interaction environment, which can promote a natural, convenient, and friendly experience as communication between people (Zhang et al., 2015; Xie et al., 2019).

In general, there are two ways to recognize emotion, i.e., through non-physiological and physiological signals. Non-physiological signals (such as facial expressions, speech, gestures, etc.) can be artificially controlled (Yao, 2014). However, physiological signals (such as electroencephalogram (EEG), electrocardiograph (ECG), electromyography (EMG), magnetoencephalography (MEG), functional near-infrared spectroscopy (fNIRS) etc.) can show reliable and natural emotions without subjective control (Cai et al., 2018). EEG and MEG are the types of electrical signal produced by the brain that provides very useful information relating to emotional activity of the brain. Moreover, EEG and MEG have good temporal resolution and both are non-invasive. Some researchers (Xing et al., 2019; Sun et al., 2020) found that the MEG signal and fNIRS signal can realize emotional recognition, and could obtain the accuracy of high binary classification. However, MEG mainly reflects the inner structure of the brain. Nevertheless, EEG is used to check the function of the brain and mainly through brain waves reflecting the mood of the brain. As a result, EEG-based emotion recognition methods have become popular in current research.

Currently, traditional machine learning methods (Yoon and Chuang, 2013; Li et al., 2018) can effectively recognize emotions but require manual feature extraction and only consider the independence of a single feature in time or space. The two-dimensional convolutional neural network (2D-CNN) of deep learning can solve these problems. However, emotion recognition requires taking into account not only the time dependence between data points but also the spatial relevance between different electrodes of EEG signals (Yea-Hoon et al., 2018). In contrast to 2D-CNN (Mei and Xu, 2017), the emotion recognition method based on three-dimensional convolutional neural networks (3D-CNN) can meet these needs (Salama et al., 2018; Zhao et al., 2020). The 3D-CNN models can automatically extract spatiotemporal features. The existing emotion recognition model has achieved high accuracy, while most researchers believe that multiple smaller-scale kernels have the ability to increase non-linear expression more than a larger kernel. Therefore, how to define the convolutional kernel size in the convolutional network is still an interesting topic in emotion recognition research.

In this paper, we propose a four-class emotion recognition method based on a multiscale convolutional kernel 3D network, in which EEG-based emotional states can be efficiently recognized. First, we located the spatial position of the EEG signal electrode according to the 10–20 system diagram, the positional relationship between the positioning electrodes, and retained the spatial information of the EEG. The emotional recognition model based on three-dimensional EEG is generally used in the size of a consistent convolutional kernel. However, this paper attempts to use different smaller sizes of convolutional kernels, which are expected to increase the non-linear features of the data and the amount of data available. In addition, we join the double linear convolutional structure to the emotion recognition network model (ERN), and the EEG data are analyzed in parallel, thereby obtaining efficient recognition results.

According to existing studies, most researchers have applied two lengths of time windows, i.e., 1 s (1 s) and 2 s (2 s). To find the most suitable time window length for the ERN model, we compare the classification performance of 1 and 2 s time window lengths. The experimental results have shown that multiscale convolutional kernels with suitable time window lengths are more effective, which can improve the accuracy for emotion recognition. In summary, the main contributions of this paper are as follows. (1) We optimized the original dataset, designed a repositioned electrode topology, and constructed a 3D dataset for the model. (2) We enriched the emotion recognition method based on EEG and constructed a multiscale convolutional kernel 3D-CNN model to achieve more efficient emotion recognition performance.

The paper is structured as follows: related research is discussed, followed by consideration of the methodology adopted in our work. Experimentation and evaluation are addressed, and a discussion with results derived from the experimentation is presented. The paper closes with concluding observations and consideration of future work.

## Related work

Currently, a variety of traditional machine learning-based emotion recognition methods have been documented in the literature, which also confirms the effectiveness and accuracy of traditional machine learning on emotion classification. However, machine learning-based emotion recognition methods require the specifically detailed design of classification models and manual extraction of temporal or spatial emotion features of EEG signals. For example, the traditional classifiers used in the literature include the support vector machine (SVM), and k-nearest neighbors (KNN) (Jenke et al., 2014). However, the use of traditional machine learning requires the manual extraction of relevant emotion features, limited to the temporal or frequency domains, with domain knowledge barriers and timeliness problems.

Emotion recognition methods based on deep learning can solve these problems. The deep network can extract different types of features at the same time, with the advantages of automatic detection features, and solve the dependence of artificial feature extraction. For example, Lin et al. (2017) proposed an emotion recognition method based on CNN, converting EEG data from the signal format into an image format containing time domain and frequency domain information, combined with the characteristics of other physiological signals, which were input into the pretrained AlexNet (Krizhevsky et al., 2012) network model for emotion recognition. Kwon et al. (2018) also proposed a sentiment classification method for extracting features based on the 2D CNN model, which were preprocessed before convoluting EEG signals by wavelet transformation considering both the time and frequency domains to improve the recognition performance.

In addition to CNN model, there are other methods effectively identify emotions. Liu et al. (2018) proposed that the Residual Network-50 (ResNet-50) model can automatically learn deep semantic EEG information and classify the new features of the fusion of linear-frequency cepstral coefficients (LFCC). Xing et al. (2019) used a stack auto-encoder (SAE) to build and solve the linear EEG mixing model and the emotion timing model based on the long short-term memory recurrent neural network (LSTM-RNN). Liu et al. (2020) proposed a capsule network based on the multi-level feature boot, which can recognize multi-electrode EEG emotions. In the same year, Tao (Tao et al., 2020) proposed a convolutional recursive neural network (ACRNN) based on the attention mechanism, which can extract more discriminant features from the EEG signal, and improve the accuracy of emotional identification.

However, these models ignore the spatial structure of the EEG and the variations and distortion of the electrodes in each dimension. With the study of deep learning neural networks, methods for extracting spatiotemporal features have been proposed. Yang et al. (2018) implemented a hybrid neural network integrating a CNN and a recurrent neural network (RNN) so that network models could extract and integrate spatiotemporal features. Wang et al. (2018) proposed a simple and efficient preprocessing method that converts multiple electrodes of EEG signals into electrode topological maps containing topological location information. An et al. (2021) proposed an EEG emotion recognition algorithm based on 3D feature fusion and a convolutional auto-encoder (CAE). Zhao et al. (2020) proposed a 3D-CNN model to automatically extract the spatiotemporal features of EEG signals, introduced the preprocessing method for baseline signal and electrode topology relocation, compared the performance of the 2D convolutional kernel and 3D convolutional kernel in detail, and showed that the 3D-CNN model was more advantageous.

Different convolutional network recognition models set different convolutional kernel sizes, while most researchers (Szegedy et al., 2016) believe that multiple smaller scale kernels have the ability to increase non-linear expression more than a larger kernel. The different sizes of kernels in the network can increase the number of features that can be used and improve model performance. In this paper, we propose a four-class emotion recognition method based on a multiscale convolutional kernel 3D network, in which EEG-based emotional states can be efficiently recognized. The experimental results on the DEAP and SEED-IV datasets show that the proposed model has preferable performance than the other existing models in terms of recognition accuracy.

## Materials and methods

**FIGURE 1:**
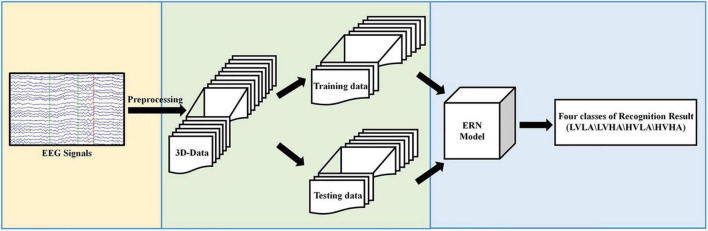
Overview of the proposed approach.

The experimental process proposed in this paper is shown in Figure 1. In Figure 1, first, the original EEG signals are preprocessed. Then, the preprocessed data are converted from the 2D form to the 3D format and divided into two kinds of datasets: training data and testing data. Finally, we input preprocessed data and evaluated our ERN model by the recognition results of the four classes of labels.

### Processing

To improve the recognition accuracy, it is necessary to preprocess the raw EEG data. First, the brain electrode data are selected and subsampled, and the noise artifacts are removed using a bandpass frequency filter of 4.0–45.0 Hz. A preprocessing method for the baseline EEG signal will affect the recognition result (Yang et al., 2018). Therefore, the specific practice of the data baseline signal processing is as follows. First, the first 3 s of baseline signals are extracted from all electrodes c of a single subject, and then cut into a fragment of the N-section fixed length L, thereby obtaining the N × C × L matrix. Then, calculate the average of this N × C × L matrix, obtain the Z matrix, and the structure of the Z matrix is C × L. The last 60 s of the signals are divided into M fragments to obtain the matrix of M × C × L. Then the matrix of M × C × L subtracts the average matrix Z. N and M are the number of data segments, C is the number of electrodes, and L is the data length. This calculation step can obtain all the data of a single subject and be repeated, and we can obtain all the pretreatment data.

The 32 electrodes of the EEG signals in the dataset are repositioned to a 2D electrode topology based on the International 10–20 System Diagram to acquire the spatial information of the EEG. During the recognition of emotional type based on EEG, Zhong et al. (2020) confirmed that both the position of signals acquisition and the interaction of EEG electrode position are conducive to improving the accuracy of emotion recognition based on EEG signals. Therefore, to retain the spatial information of the EEG, we located the spatial position of the EEG signal electrode according to the 10–20 system diagram (the positional relationship between the positioning electrodes), and retained the spatial information of the EEG. Based on the topological location information of the vulnerable electrodes during the original EEG emotion analysis experiment, Zhong and An (Zhong et al., 2020; An et al., 2021) proposed a solution by repositioning the 32 electrodes of the EEG signals in the dataset to the 2D electrode topology based on the international 10–20 system diagram, thereby preserving spatial information among electrodes. In this paper, 1-dimensional (1D) electrodes of the obtained dataset are repositioned into a 2D electrode topology.

**FIGURE 2:**
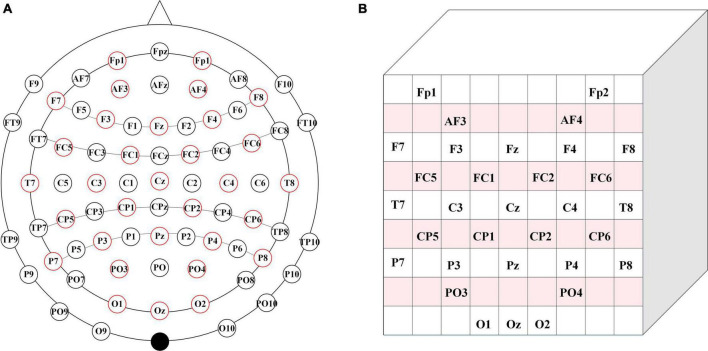
Brain electrode distribution diagram. **(A)** Brain electrode distribution in 10–20 system diagram. **(B)** The corresponding matrix.

As shown in Figure 2A, we choose the 32-electrode EEG data of the dataset, which is located in the International 10–20 system diagram (Sharbrough et al., 1991). According to the farthest distance between the two electrodes in Figure 2A, we set the size of the two-dimensional matrix and the size of the two-dimensional matrix is 9 × 9. Then, the selected 32 EEG signals are mapped to the 9 × 9 matrix. In Figure 2B, the positioning of each electrode is located based on the positional relationship between the various electrodes in Figure 2A. The blank position is represented as a topological position of the unselected physiological signal. Therefore, unused topologies are set to zero in the 9 × 9 matrix, and the matrix is normalized.

### Model

We design the multiscale convolutional kernel 3D-CNN model based on the final obtained 3D EEG dataset. The reasons for the use of this network are as follows: First, the advantage of the convolutional network (Zeiler and Fergus, 2014b) is that it can calculate the eigenvalue rather than the original value with no need for the accurate mathematical expression between inputs and outputs. Second, the convolutional network can avoid the problem of gradient loss when reverse propagation occurs in the BP neural network. The CNN can be trained in parallel, which reduces the complexity of the network. In particular, the network can directly input multi-dimensional data directly, which avoids the complexity of data reconstruction during feature extraction and classification. The flexibility of the three-dimensional convolutional kernel is higher than that of the two-dimensional convolutional kernel, which helps to learn the advanced representation of learning information (Tran et al., 2015). The controllable range of the three-dimensional convolutional kernel is expanded to the spatial domain, which can utilize the interaction between the electrodes and increase the identification ability of the model.

**FIGURE 3:**
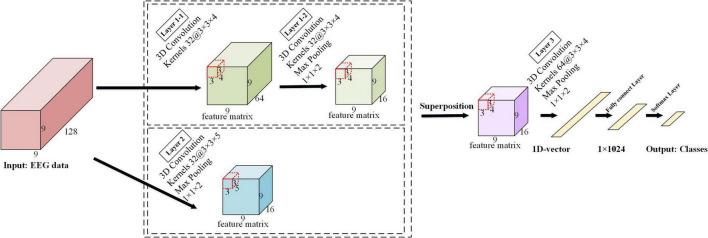
The framework of the ERN model.

The detailed architecture of the ERN model is shown in Figure 3. The architecture of this 3D-CNN model consists of three convolutional layers, with the first convolutional layer implemented in parallel with the second convolutional layer in the model. The kernel size is 3 × 3 × 4 in the first convolutional and the third convolutional layers, where spatiotemporal features are generated by the local spatial topology of 3 × 3 and fragments of the temporally sampled point 4. The kernel size is 3 × 3 × 5 in the second convolutional layer and combines advanced spatial features via a local spatial topology of 3 × 3 and a temporal sampling point of 5. The first convolutional layer and the second convolutional layer are used to calculate the feature images, and the obtained feature images are superimposed to obtain a new feature image. Multiple 3 × 3 kernels have more non-linear functions than a larger convolutional kernel, which increases the non-linear expression and makes the judgment function more efficient. Selecting more small convolutional kernels favors more accurate emotion recognition. Under the conditions of ensuring the same kernel, the depth of the network is improved, the parameters of the model are reduced, and the effect of the neural network is improved to some extent.

**FIGURE 4:**
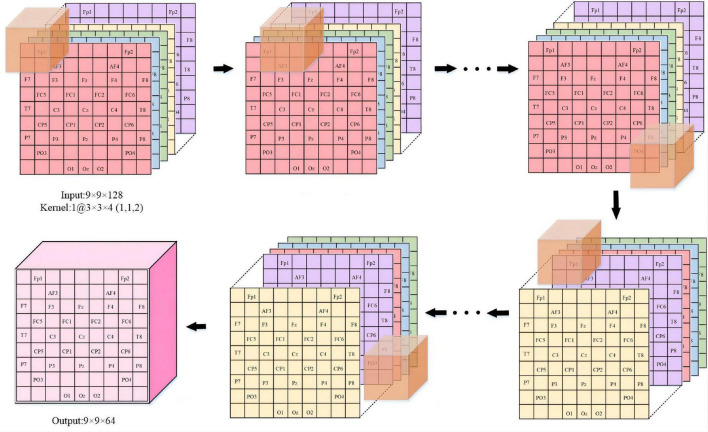
The feature representation learned in ERN. (Overview of the proposed approach detailed convolutional operations).

We provide a detailed description in Figure 4 to visualize the feature representation learned by the hidden layers of the ERN model. Taking 3 × 3 × 5 as an example, the step size of the convolutional kernel is 1, the format of the input data is 9 × 9 × 128, and the resulting feature format is 9 × 9 × 64. The figure demonstrates the detailed process of the convolutional operation. Each convolutional kernel is used to compute features and moves with a fixed step on the dataset. We can calculate the size of the features according to the equation (1). *I_n_*(*n = 1,2,3*) is defined as the size of the input convolutional layer data, *O_n_*(*n = 1,2,3*) is the size of the output convolutional feature, *K_n_*(*n = 1,2,3*) is the convolutional kernel size, n is one of the three dimensions, *N* is the number of convolutional kernels, S is the moving step of the convolutional kernel, and *P* is padding value; this article set *P* is 1. The size of the three dimensions of the feature shown in Figure 4 can be calculated separately by the equation (1). Equation (1) can calculate the size of the 3D features map.


(1) $$O_{n}=\frac{I_{n}-K_{n}+2P}{S}+1$$

A 3D maximum pooling layer is set behind each convolutional layer, and the kernel size is 1 × 1 × 2. The maximum pooling layer (Scherer et al., 2010) is used to extract the features more efficiently here; it can reduce the quantity of data on the time dimension and improve the robustness of the extracted features and provides a better generalization. The first maximum pooling layer and the second maximum pooling layer further squeeze the extracted spatiotemporal features to generate advanced high-level spatiotemporal features. The last pooling layer is followed by a fully connected layer, and the Softmax layer is deployed as the output. In the experiment, the input data size of the model is 9 × 9 × 128, where 9 × 9 is the 2D electrode topology and 128 is the number of continuous-time sampling points for one treatment. The number of feature maps for the last convolutional layer is 64, passing the 64 feature maps to the fully connected layer, which maps the input as vectors. The N in its output represented the number of labels in the task. The empty is set to zero in each convolutional layer to prevent the loss of information from the input data, and the ReLU activation function is used after each convolutional layer.

## Experiment

We test the model in the public databases DEAP (Koelstra et al., 2012) and SEED-IV (BCMI, 1994). We use the PyTorch framework (Chaudhary et al., 2020) to implement this model and deploy it on the GeForce RTX 3060. The learning rate is set to 0.001 with the Adam AdaDelta Optimizer, and the probability of the dropout operation is set to 0.6. We use 10-fold cross-validation to evaluate the performance of the ERN model. The average accuracy of the 10-fold validation processes is taken as the final result.

### Processing

#### DEAP dataset

The multimodal DEAP dataset is an open multimodal standardized dataset used to study the analysis of human emotional states. The dataset includes the 32 electrodes of EEG signals and the 8 electrodes of peripheral physiological signals when subjects watch music videos. After watching a video, subjects scored each video based on four psychological scales of arousal, valence, liking, and dominance. We select 32 electrodes of EEG signal data from the dataset for the analysis of the human emotional state.

The preprocessing step is as follows: First, the data are downsampled from 512 to 128 Hz, and then a bandpass frequency filter of 4.0–45.0 Hz to remove noise artifacts. At this time, the processed dataset data structure is 40 × 32 × 8,064 (video number × EEG electrode number × signal data), of which 8,064 signal data contained 384 baseline signals. The DEAP dataset is divided into two parts, as shown in Table 1. The data matrix refers to the EEG of 40 electrodes observed when each subject watched music videos. The label matrix refers to the four types of labels after each subject watches videos: arousal, valence, dominance, and liking.

**TABLE 1:** The DEAP dataset and SEED-IV dataset.

Matrix name	Matrix structure representation
DEAP Dataset	
*Data*_40 × 40 × 8,064_	*Data*_*video* × *channel* × *fixedpointintime*_
*Label*_40 × 4_	*Label*_*video* × *value*_
SEED-IV Dataset	
*Data*_15 × 62 × *_	*Data*_*video* × *channel* × *fixedpointintime*_
*Label*_15 × 1_	*Label*_*video* × *value*_

In this dataset, each video of each stimulus is 60 s so that the first 3 s is the baseline signals of the unstimulated, and the last 60 s is the signals of the stimulus in the 63 s signals of each stimulus trial. Therefore, we need to carry out baseline signal processing for each trial signal after preprocessing. The processing step is as follows: For each trial signal (32 × 8,064), cut the baseline signal (32 × 384) of the first 3 s to 3 segments (32 × 128) and calculate the mean value of the baseline signals (32 × 128). Then, the signal data of the last 60 s are cut into 60 segments (32 × 128), and the mean of the baseline signal is substracted and merged to obtain the processed signal (32 × 7,680). Next, each electrode needs to be repositioned to the two-dimensional topological location to learn the spatial properties of the data. 128, 384, 7,680, and 8,064 are the time points and 32 stands for the number of electrodes. To extract the spatiotemporal features, the EEG data are mapped into a 9 × 9 matrix based on the International 10–20 system diagram. Finally, the matrix is cut into fragments with a length of 1 s (9 × 9 × *), and the 3D electrode topology was obtained (7,680 9 × 9 × *), of which the symbol “*” represents the size of the time window and 7,680 represents the number of matrixes.

Specifically, for the selected labels, the valence describes the degree of pleasure associated with the stimulus, represented by continuous values ranging from 1 (negative) to 5 (neutral) to 9 (positive). Arousal represents the degree of waking to the stimulus with the same range, with 1 and 9 indicating negative and positive, respectively. As shown in Table 2, we set the distribution of 4 label values based on EEG arousal and valence markers: low valence vs. low arousal (LVLA), low valence vs. high arousal (LVHA), high valence vs. low arousal (HVLA), and high valence vs. high arousal (HVHA). As shown in Table 2, we set the values of the four types of labels based on arousal and valence and set 5 as the threshold. After processing, the label structure is 40 × 1 (number of videos × label value).

**TABLE 2:** Label values in the DEAP.

Label	LVLA	LVHA	HVLA	HVHA
Valence	≤ 5	≤5	> 5	>5
Arousal	≤ 5	> 5	≤ 5	> 5
Value	0	1	2	3

#### SEED-IV dataset

We also use the SEED-IV dataset as a standardized dataset to study the model recognition performance of this paper, which is a well-formed multimodal dataset for emotion recognition. In the SEED-IV dataset, a total of 15 subjects participated in the experiment. For each subject, the test, respectively, was performed on three different days and each test contained 24 trials. In each trial, his or her EEG signals are saved when the subject watches each film clip.

The preprocessing step is as follows: First, the same 32-electrode EEG data as DEAP in the SEED-IV dataset were selected to analyze the human emotional state. Then, the data are downsampled from 1,000 Hz to 128 Hz using a bandpass frequency filter of 4.0–45.0 Hz to remove noise artifacts. The SEED-IV dataset is divided into two parts, as shown in Table 1. The data matrix refers to the physiological data of 62 electrodes observed that include the EEG signal and peripheral physiological signal. In the data matrix, the length of the movie clips resulted in the different lengths of the EEG data in each trial. The label data matrix refers to the four types of labels played when a subject watches the film clips: happy, sad, neutral, and fear.

In the SEED-IV dataset, the length of the data varies in each trial. Therefore, we need to select the data length suitable for the model after preprocessing in each stage of each subject and obtain 15 matrixes, each with 32 rows and 128 columns (32 × 128), and 15 is the number of movie clips. In each 32 × 128, the 32 is the number of EEG electrodes and 128 is the data of 1 s. Then, 32 EEG electrodes are mapped into the matrix with 9 rows and 9 columns, and 15 3D-matrixes (9 × 9 × 128) are obtained. Finally, we combine the data from three stages of 15 subjects and obtain 675 (15 × 3 × 15, subject number × stage number × video number) segments of the total dataset (9 × 9 × 128). After processing, the label structure is 15 × 1 (number of videos × label value).

### Optimal time window in model

We test and select the time window length more suitable for the experiment to obtain the best recognition result of the model and apply the 1 s time window. To improve the accuracy of the data in the experimental input model, one of the solutions of this paper is the application of the time window. The available window size is not necessarily fixed, it can constantly expand until certain conditions are met, it can be constantly reduced until a minimum window to meet the conditions is found, and it can be a fixed size.

**FIGURE 5:**
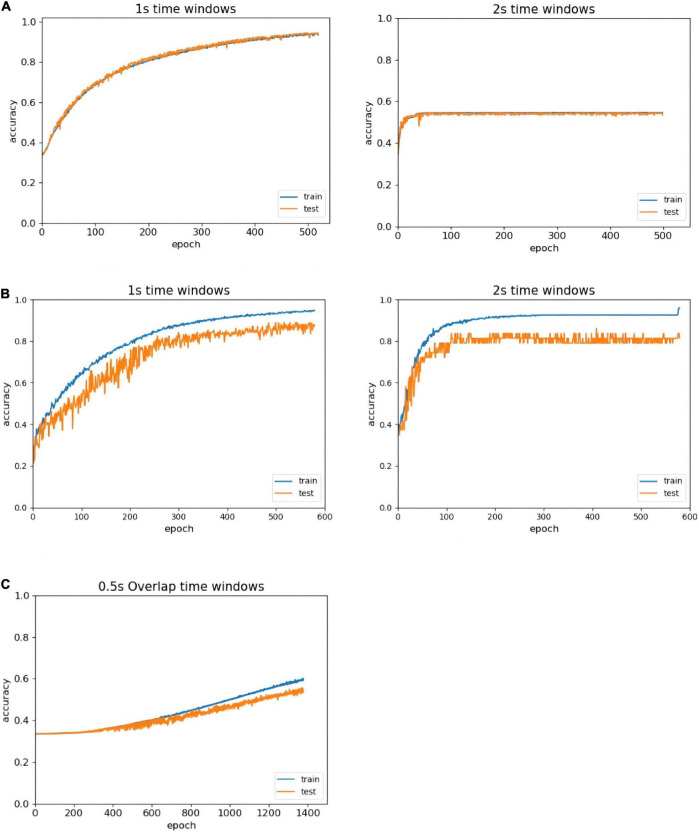
Accuracy comparison of different time window lengths. **(A)** Comparison of different time windows in the DEAP without overlapped time window. **(B)** Comparison of different time windows in the SEED-IV without overlapped time window. **(C)** The recognition of overlapped window in the DEAP.

In the literature, people (Zhao et al., 2020) confirmed that the average classification accuracy of a 1 s period based on EEG was superior to other periods, and selected the 1 s length as the most appropriate time window length. However, someone (Candra et al., 2015) believed that a length of 2 s was the most appropriate time window length. To address this problem, we compare all EEG classification performances at two different time window lengths: 1 and 2 s. In Figure 5, the left side of the figure shows the recognition result trend of 1 s time window data, and the right side of the figure shows the recognition result trend of 2 s time window data. The classification accuracy of the 1 s time window is better than that of the 2 s time window with the same number of iterations, and no overfitting occurs in the first 500 epochs. Therefore, we compare the results of the different time windows, select the time window that is more suitable for the model, and improve the accuracy of the experiment.

Since we have chosen the more suitable size of the time window for the ERN model, and the next step is the analysis of whether the ERN model needs to set the overlapping window. Taking the DEAP dataset as an example, we set 0.5 s overlapping windows to process the dataset. Depending on the identification result after using the overlapping window, there may be multiple problems. First, the baseline processing method requires each second of data to subtract the mean of the baseline data. This method can disconnect the time continuity of data and eliminate the advantage of high time resolution. Second, the amount of data doubled after using the 0.5 s overlapping time window. About another data set SEED-IV, the data length is 128 after the process of downsampling. The pre-processed data in the SEED-IV have high time continuity, so there are no setup 0.5 s overlapping windows to process this dataset. The overlapping window processing generates a large amount of available data. But in the same number of iteration, the recognition efficiency by overlapping window processing is much lower as shown in Figure 5C. To achieve out high-efficiency recognition accuracy, we have not used overlapping windows.

## Results and discussions

### Accuracy

To estimate the accuracy of the emotion recognition for the ERN model, we conducted a comparative analysis that compared the results obtained from our proposed approach applied over the DEAP and SEED-IV datasets with other methods. All of the classification procedures were conducted under 10-fold cross-validation (Yu et al., 2015), and the average value of 10 accuracies, *F*_1_ values and AUC (the area under the ROC curve) values were calculated as the evaluation standard of model accuracy. In the process of training, the optimal weight parameters are obtained and set by random training data to avoid the overfitting problem caused by optimization.

Table 3 shows the 10-fold cross-validation accuracy in the DEAP and SEED-IV datasets. The 2D-CNN only utilizes high features of EEG time resolution in EEG and neglects space information. In contrast to 2D-CNN (Mei and Xu, 2017), the emotion recognition method based on three-dimensional convolutional neural networks (3D-CNN) can meet the need (Salama et al., 2018; Zhao et al., 2020) for spatial information. Not only does the 3D-CNN extract the time characteristics based on the 1 s time window, but it can also obtain the spatial feature between the electrodes. The previous literature report and the ERN model experiments in this article show that our model has the ability to improve the accuracy of EEG-based emotion recognition.

**TABLE 3:** Ten-fold cross-validation accuracy in the DEAP and SEED-IV datasets.

Fold ID	DEAP	SEED-IV
Fold 1	94.78%	88.56%
Fold 2	95.83%	85.07%
Fold 3	95.42%	92.54%
Fold 4	97.08%	94.78%
Fold 5	93.87%	94.04%
Fold 6	96.67%	82.29%
Fold 7	96.78%	85.46%
Fold 8	93.75%	89.48%
Fold 9	95.85%	90.78%
Fold 10	96.67%	92.47%
Mean	95.67%	89.55%

In Table 4, the average accuracy of our model for four emotion classes is up to 95.67% in DEAP and 89.55% in SEED-IV datasets, which is higher than previously reported models where 93.72 and 87.71% accuracies were achieved. It is known that the model based on deep learning proposed by Qiu et al. (2018) and Zhao et al. (2020) currently has the best performance in the DEAP dataset and SEED-IV dataset. However, the results of the ERN model are approximately 1.95 and 1.84% higher than those models. Compared with the CNN model (Mei and Xu, 2017; Salama et al., 2018; Tao et al., 2020; Zhao et al., 2020; Yin et al., 2021) and compared with other methods (Zangeneh et al., 2019; Song et al., 2020; An et al., 2021; Li S. et al., 2021) in the DEAP dataset, our model adopts a simpler and more efficient structure and has the best performance. Our model has higher speed efficiency and better identification performance than other models (Qiu et al., 2018; Zheng et al., 2019; Acharya et al., 2020) in the SEED-IV dataset.

**TABLE 4:** Comparison of ERN model with previous studies.

Research	Year	Method	Accuracy
DEAP dataset			
Mei and Xu	2017	2D-CNN	73.10%
Salama et al.	2018	3D-CNN	88.49%
Zangeneh et al.	2019	HcF+KNN+MSVM	86.01%
Song et al.	2020	DGCNN	90.4%
Zhao et al.	2020	3D-CNN	93.53%
Tao et al.	2020	ACRNN	93.72%
Li S. et al.	2021	The binary gray wolf optimization algorithm+SVM	90.48%
Yin et al.	2021	GCNN+LSTM	90.53%
An et al.	2021	3D Feature Fusion+CAE	90.76%
Our model	2021	3D-CNN	95.67%
SEED-IV Dataset			
Zheng et al.	2015	DBN	86.08%
Qiu et al.	2018	CAN	87.71%
Zheng et al.	2019	EmotionMeter	85.11%
Acharya et al.	2020	LSTM	87.22%
Our model	2021	3D-CNN	89.55%

For classification, cross-validation is not effective protection against overfitting or overhyping. It would be better to use techniques such as lockboxes, blind analyses, pre-registrations, or nested cross-validation to limit overhyping. We use the lockbox (Hosseini et al., 2020) approach to determine whether overhyping has occurred in the CNN model. The lockbox approach is a new technique that can be used to determine whether overhyping has occurred. The lockbox is accessed just one time to generate an unbiased estimate of the model’s performance. In the DEAP and SEED-IV datasets, 90% of the data are set aside in the hyperparameter optimization set and the remaining 10% of the data are set aside in a lockbox. With the 10-fold cross-validation approach, the hyperparameters in the ERN model can be iteratively modified on the hyperparameter optimization set. When the average accuracy in the model is good enough, the model is tested on the lockbox data.

As shown in Table 5, the training result on the hyperparameter optimization set and the testing result on the lockbox set are 98.59 and 95.67% in the DEAP, 93.05 and 89.55% in the SEED-IV. According to the identification result, we can obtain the following conclusions. The theta band is in the state of sleep and a less responsive emotional state, so the recognition rate of emotion is lower than that of the other three waveforms. The excitement state of alpha, beta, gamma waveforms increased successively, so the recognition accuracy of emotion is higher.

**TABLE 5:** Comparison of ERN model with different bands.

Modality	Train result (%)	Test result (%)
EEG signals (DEAP)		
Theta	87.81	84.40
Alpha	92.47	88.74
Beta	97.01	90.91
Gamma	94.39	88.31
EEG	98.59	95.67
EEG signals (SEED-IV)		
Theta	83.08	82.09
Alpha	90.48	86.57
Beta	85.65	85.07
Gamma	92.45	88.06
EEG	93.05	89.55

### *F1* and *ROC*

We also calculated the *F*_1_ value and *ROC* value to analyze the performance of the ERN. In Formulas (2) and (3), *F*_1_ is the unweighted average of multiple categories of *F*_1_*m*__ (*m* = 0,…, C, C = 3), where *m* means four classes of emotion: LVLA, LVHA, HVLA and HVHA. *F*_1_*m*__ is calculated from *Precision*_*m*_ and *Recall*_*m*_, and *Precision*_*m*_ and *Recall*_*m*_ are calculated from *FN*_*m*_, *TP*_*m*_, *TN*_*m*_ and *FP*_*m*_. In Formulas (4) and (5), *FN*_*m*_ and *TP*_*m*_, represent the number of (incorrectly) recognized samples of a certain category, *FP*_*m*_ and *TN*_*m*_, represent the number of (incorrectly) recognized samples of other categories except the *m*-th emotion category. According to Formula (6) and Formula (7), the true positive rate (*TPR*) and false positive rate (*FPR*) are calculated by *FN*_*m*_, *TP*_*m*_, *TN*_*m*_ and *FP*_*m*_, and the *ROC* curves of the model are calculated to obtain *AUC*_*m*_ (the area under the *ROC* curve) of each category.


(2) $$F_{1}=\frac{1}{C}\sum_{m=0}^{C}F_{1_{m}}$$

(3) $$F_{1_{m}}=\frac{2\times Precision_{m}\times Recall_{m}}{Precision_{m}+Recall_{m}}$$

(4) $$Precision_{m}=\frac{TP_{m}}{TP_{m}+FP_{m}}\;$$

(5) $$Recall_{m}=\frac{TP_{m}}{TP_{m}+FN_{m}}$$

(6) $$TPR=\frac{TP_{m}}{TP_{m}+TN_{m}}$$

(7) $$FPR=\frac{FP_{m}}{TN_{m}+FP_{m}}$$

**FIGURE 6:**
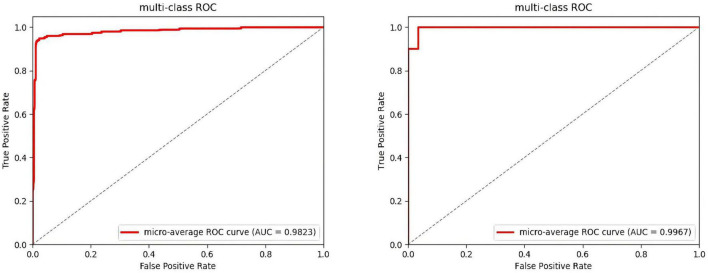
Average ROC of the four-classes. (The ROC of the DEAP in the left, the ROC of the SEED-IV in the right).

After several iterations, the values of the four classes are shown in Table 6. In the DEAP dataset, the LVLA, LVHA, HVLA, and HVHA values are 97.51, 98.62, 98.03, and 98.76, respectively. In the SEED-IV dataset, the LVLA, LVHA, HVLA, and HVHA values are 99.92, 98.97, 99.94, and 99.85, respectively. As shown in Figure 6, the solid red line is the average ROC curve of the four categories, and the average AUC is 98.23 for DEAP and 99.33 for SEED-IV.

**TABLE 6:** *F*_1_ values and AUC values of the four-class.

Dataset	*m*	0	1	2	3	Mean
DEAP	*F*_1_*m*__	94.82	95.06	94.52	96.742	95.29
	*AUC*_*m*_	97.51	98.62	98.03	98.76	98.23
SEED-IV	*F*_1>_*m*__	95.652	96.97	92.683	85.714	92.75
	*AUC*_*m*_	99.92	98.97	99.94	99.85	99.67

**FIGURE 7:**
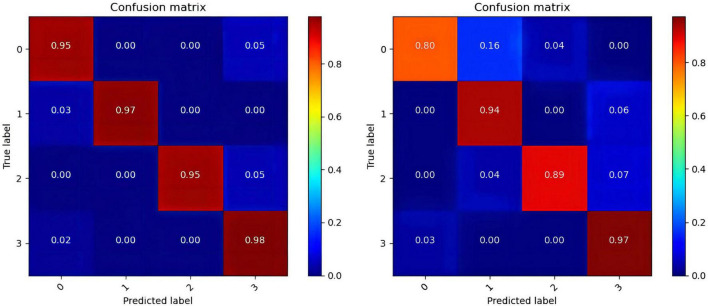
The confusion matrixes of datasets. (The confusion matrix of the DEAP in the left, the confusion matrix of the SEED-IV in the right).

In the operation process, mass test data and the large threshold distance between the two samples result in the ROC curve not being smooth in Figure 6. The higher values of the four categories indicate that the model constructed in this paper has better performance, among which the data of the fourth category (HVHA) have higher identifiability. Each column of the confusion matrix represents the predictive category, and the total number of each column indicates the number of data predicted for this category. Each line represents the real category of the data, and the total number of each line of data represents the number of data instances of the category in Figure 7. The matrix verifies that our model is stronger than others in predicting complex labels.

From our experimental results shown in Tables 4–6, Figures 6, 7, it can be seen that our results are superior to those of previous studies reported in the literature over the same EEG datasets from DEAP and SEED-IV. There are 3 possible reasons. (1) We conduct a simple and efficient preprocessing method, including data baseline signal processing, EEG electrode topological mapping, and 1 s time window length selection. (2) A 3D convolutional structure is a necessary technique to study emotional recognition based on EEG signals, as this structure can identify space information of the electrodes to quickly extract spatiotemporal features. (3) The multiscale convolution kernel not only reduces the computation of the model but also improves the identification ability of the model because multiple smaller scale kernels have the ability to increase non-linear expression more than a larger kernel. These all enhance the operating efficiency and improve the recognition performance of the ERN model.

### Fisher

The parietal lobe of the human brain and non-human primate brain have been associated with attention based on the evidence of clinical and physiological (Joseph, 1990). Studies in literature show that the lateral Intraparietal area (LIP) plays an independent role in target selection and visual attention generation (Simon et al., 2002). These findings can be validated by the distribution of Fisher’s selected EEG electrodes. We score the 32 electrodes of the applied dataset using the *Fisher* (Li R. et al., 2021) scorer and rank the 32 electrodes from highest to lowest to obtain the top 8, top 16 and top 24. In Formula (8), μ_*m*,*v*_ is the average of each electrode of the *m*-th class, μ_*v*_ is the average of each electrode, σ_*m*,*v*_ is the variance of each electrode of the *m*-th class and *n_m_* is the number of samples of the *m*-th class (*m* = 0,…,M, M = 3).


(8) $$Fisher=\frac{\sum_{m=0}^{M}n_{m}{\left(\mu_{m,v}-\mu_{v}\right)}^{2}}{\sum_{m=0}^{M}n_{m}\sigma_{m,v}^{2}}$$

We can sort the impact sequence of different electrodes based on emotions by the recognition results. According to the output results of the ERN model, we can study the recognition performance of the model for many EEG electrodes. Table 7 shows that the emotion recognition results of different quantities of electrodes in the sort. Experimental results showed that there is a 4.7% difference between the data of the first 8 electrodes and the whole dataset in DEAP and a 5.97% difference between the data of the first 8 electrodes and the whole dataset in SEED-IV. This indicates that the model can quickly obtain high recognition performance with less EEG electrodes data. We can use the ERN model for portable emotional identification (Cai et al., 2018) based on EEG and the model meets simple and fast needs. According to the identification results of the first 8, 16 and 24 electrodes in the table, most of the effective EEG electrodes are distributed in the frontal and parietal lobes (such as, O2, PO4, AF3, F3, F7, FC5, FC1, and so on). Therefore, the frontal and parietal lobes have a large effect on emotional identification.

**TABLE 7:** Evaluation of recognition results based on *Fisher.*

Dataset	The number of electrodes	The name of electrodes	Accuracy
DEAP	8	O2\PO4\AF3\F3\F7\FC5\FC1\C3	90.31%
	16	O2\PO4\AF3\F3\F7\FC5\FC1\C3\T7\CP5\CP1\P3\P7\PO3\O1\Oz	92.07%
	24	O2\PO4\AF3\F3\F7\FC5\FC1\C3\T7\CP5\CP1\P3\P7\PO3\O1\Oz\ Pz\Fp2\AF4\Fz\F4\F8\FC6\FC2	93.03%
	32	O2\PO4\AF3\F3\F7\FC5\FC1\C3\T7\CP5\CP1\P3\P7\PO3\O1\Oz\Pz\Fp2\AF4\Fz\F4\F8\FC6\FC2\Cz\C4\T8\CP6\CP2\P4\P8\Fp1	95.67%
SEED-IV	8	Oz\O2\FP2\AF3\AF4\F7\F3\Fz	83.58%
	16	Oz\O2\FP2\AF3\AF4\F7\F3\Fz\F4\F8\FC5\FC1\FC2\FC6\T7\C3	85.07%
	24	Oz\O2\FP2\AF3\AF4\F7\F3\Fz\F4\F8\FC5\FC1\FC2\FC6\T7\C3\Cz\C4\T8\CP5\CP1\CP2\CP6\P7	86.57%
	32	Fp1\FP2\AF3\AF4\F7\F3\Fz\F4\F8\FC5\FC1\FC2\FC6\T7\C3\Cz\C4\T8\CP5\CP1\CP2\CP6\P7\P3\PZ\P4\P8\PO3\PO4\O1\Oz\O2	89.55%

### Ablation experiments

The generic dimension and volume type of the kernel have 3 × 3, 5 × 5, and 7 × 7, where a plurality of 3 × 3 stacked approximately a 5 × 5 or 7 × 7. Because the activation function is set after the convolutional layer, Krizhevsky et al. (2012) believes that the recognition capability of the model can be controlled by the volume of kernel. Multiscale small kernel subscriptions have diversely increased the network capacity so that the decision function is more distinguished for different categories.

We assume that the size of the 3D convolutional kernel is M × N × K. When using a 3D kernel, it can be divided into three steps: First, complete the convolution of the M × 1 × 1 content; second, complete the convolution of the 1 × N × 1 content; finally, complete the convolution of the 1 × 1 × K content. The total convolution process can increase the non-linear expression of the model because the local convolution of each small step is completed and pass through the non-linear function.

We can clearly see that the entire process has three non-linear transformations. Therefore, the non-linear characteristics of the results eventually increase, making the decision function more decisive and helping the model increase the accuracy of emotion recognition. The size of conv3 × 3 (the size of convolutional kernel is 3 × 3) compared to conv5 × 5 and conv7 × 7 significantly reduces the number of parameters. Simonyan and Zisserman (2014) replaces a conv7 × 7 with three conv3 × 3, which is considered to further decompose the characteristics mentioned by the 7 × 7 larger volume kernels. The Regularization of the multiscale small kernel can improve the model performance.

In addition to the small conv3 × 3, there is the small conv2 × 2. However, Zeiler and Fergus (2014a) studies conv2 × 2 and is unable to find the central point of the convolution, which causes the characteristics of the padding process to constantly offset. As the number of layers deepens, conv2 × 2 makes the distance of feature offset increasingly obvious. Thus, this paper expects to apply the multiscale convolutional kernel to increase the feature amount of the model calculation and improve the model recognition.

According to the above research results, this paper takes the types of convolutional kernels of conv3 × 3. The same dataset is carried out by different types of kernels and each classification result is compared. Conv3 × 3* represents the multiscale kernel, and conv3 × 3 represents the same-scale kernel. Table 8 shows that the multiscale small kernel subscriptions diversely increase the network capacity so that the decision function is more distinguished for different categories.

**TABLE 8:** Comparison of different kernels.

Kernel Size	DEAP (%)	SEED-IV (%)
Conv3 × 3*	95.67	89.88
Conv3 × 3	92.88	85.13

*represents the multiscale kernel.

## Conclusion

In this paper, we have presented the ERN model which uses the multiscale 3D-CNN to recognize emotions based on EEG. We obtain the optimal parameters through random training data and design experiments to compare the promotion classification performance at different time windows. Then, based on the 1 s time window dataset with better classification performance, an effective multiscale convolutional kernel 3D-CNN model based on EEG signals is implemented to simultaneously extract spatial and temporal features, and achieves higher accuracy of emotion recognition.

In the comparative analysis using the EEG signals in the DEAP and SEED-IV datasets, we have demonstrated the superior accuracy, *F*_1_, and AUC values of emotion recognition for the ERN model based on multiscale 3D-CNN. From the experimental results, we show that this model can achieve higher performance, which helps to efficiently recognize the emotional state of the subjects so that BCI technology can quickly and accurately convert the neural electrical signal into commands that can be identified by the computer, greatly improving human-machine interaction.

The limitations of the model include the exploratory interpretability of the convolutional model. The calculation process in the convolution network is similar to a black box, and it is especially difficult to understand how the method works on feature learning. If the feature representation learned by the hidden layers can be visualized, it will be more conducive to the optimization of the convolutional network.

While we have achieved superior accuracy when compared to alternative methods as discussed in this paper using EEG data, we consider that there are further potential improvements. Our projected future directions for research include addressing subject-independent emotion recognition (the model can be trained using data acquired from a limited number of participants and can be applied to a subject who has never experienced the system prior to the experiment.) used our model to conduct the fusion of multimodal signals such as EEG and EMG studies and the investigation of other methods to improve our model with respect to the recognition accuracy. In addition, we can also use the model to challenge other tasks, such as emotion recognition based on multimodal physiological signal fusion, which could facilitate the performance of real-time emotion recognition and enhance emotional experience in the field of BCI.

## Data availability statement

Publicly available datasets were analyzed in this study. This data can be found here: http://www.eecs.qmul.ac.uk/mmv/datasets/deap/ and https://bcmi.sjtu.edu.cn/~seed/index.html.

## Author contributions

YS and ZZ designed this project, carried out most of the experiments and data analysis, and revised the manuscript. All authors analyzed the results and presented the discussion and conclusion, contributed to the article and approved the submitted version.
